# Endoscopic endonasal resection of a large tuberculum sella meningioma

**DOI:** 10.3171/2020.4.FocusVid.19981

**Published:** 2020-04-01

**Authors:** Ahmed Mohyeldin, Jayakar V. Nayak, Juan C. Fernandez-Miranda

**Affiliations:** Department of Neurosurgery, Stanford University Medical Center, Palo Alto, California

**Keywords:** meningioma, tuberculum sella meningioma, skull base, endoscopic endonasal surgery, virtual reality, surgical video, surgical anatomy

## Abstract

Over the past three decades, endoscopic endonasal surgery has unlocked new corridors to treat a wide spectrum of ventral skull base lesions. Tuberculum sella meningiomas represent one of the most ideal pathologies for ventral skull base access. Traditionally, these lesions were approached primarily through various subfrontal and frontal-lateral transcranial approaches that have unfortunately been shown to be associated with worsening visual decline postoperatively. The endoscopic endonasal approach is now being attempted by more surgeons and leverages an infrachiasmatic trajectory that provides direct access to the tuberculum sella where most of the vascular supply for these lesions can be taken early, facilitating more efficient surgical resection and mitigating the risk of optic nerve injury. Here we review a challenging case of a large (∼3 cm) tuberculum sella meningioma, encasing critical vessels off the circle of Willis and resected via an endoscopic endonasal approach. We discuss the technical nuances and relevant surgical anatomy of this approach and highlight important considerations in the safe and successful removal of these meningiomas. We show that certain tumors that appear to encase the supraclinoidal carotid artery can be fully resected via an endonasal approach with precise surgical technique and adequate exposure. Furthermore, this case illustrates the risk of injuring a key perforating vessel from the anterior communicating artery complex, called the subcallosal artery. Injury to this vessel is highly associated with tumors like the one presented here that extend into the suprachiasmatic space between the optic chiasm and the anterior communicating complex. Meticulous surgical dissection is required to preserve this perforating vessel as well as branches from the superior hypophyseal artery. Finally, we review our current closure techniques for these challenging approaches and discuss the use of a lumbar drain for 3 days to lower CSF leak rates.

The video can be found here: https://youtu.be/mafyXi5B0MA.

## Transcript

Here we present the case of a large, challenging tuberculum sella meningioma resected via an endoscopic endonasal approach. This is a case of a 24-year-old female who presented with progressive visual decline, and an MRI revealed a large meningioma compressing her optic chiasm. Preoperative visual field testing revealed minimal to no light perception in the left eye and severe compression of the optic chiasm, that revealed a right eye peripheral visual field loss (0:50).

**0:53** A preoperative CT angiogram helped us create and simulate a three-dimensional reconstruction, using virtual reality to better study the relationships the tumor made with surrounding blood vessels off the circle of Willis.

**1:12** Understanding the relevant anatomy in this location is critical to developing a successful surgery and approach. This is a picture of the sphenoid bone (1:20) projecting from a posterior to anterior perspective. The black dotted line demarcates an important anatomical landmark called the sphenoid limbus, which demarcates the level at which the optic nerves are usually seen.

**1:37** Below that is the chiasmatic sulcus where the optic chiasm is found.

**1:43** Below the chiasmatic sulcus is a bony prominence called the tuberculum sella. This is where the vast majority of these tumors (1:50) are born and where they receive their vascular supply. Moving more posteriorly, we find the sella turcica, which is where the pituitary gland lives. Beyond that, we find the dorsum sella and bilateral posterior clinoid processes. Traditional transcranial approaches to get to this area include subfrontal approaches, as well as frontal-lateral approaches, including orbital zygomatic and pterional. Unfortunately (2:20), these approaches have been associated with increased risk of visual decline postoperatively. The endoscopic endonasal approach offers direct access to the tuberculum sella and allows the surgeon to leverage an infrachiasmatic approach to the tumor and its blood supply.

**2:41** Careful review of the anatomy from an endonasal perspective helps us deconstruct the approach. Seen here is an endoscopic view of the ventral skull base (2:50) as seen through the sphenoid sinus. As depicted, the sella is surrounded by the paraclinoidal carotid arteries. Separating the carotid arteries from the optic nerve is a pneumatization of the optic strut called the lateral OCR, which stands for optocarotid recess. The tuberculum sella is seen superiorly above the sella, and separating the sphenoid limbus from the tuberculum sella (3:20) is the chiasmatic sulcus. The sphenoid limbus represents the horizon of the superior extent of the approach.

**3:30** This is a simulated representation of the exposure that is required for this approach. To achieve this, the superior and middle turbinates were lateralized and not resected. A wide post ethmoidectomy and sphenoidotomy were performed to gain access to the sinus. The face of the sella was drilled (3:50) and removed and the paraclinoidal carotids were also exposed. The tuberculum sella was drilled all the way up to the sphenoid limbus exposing the necessary dura from the inferior sellar floor all the way up to the sphenoid limbus in order to gain safe access for resection. The operative setup is depicted below; the patient’s head is placed in a Mayfield’s head clamp and positioned 180° from anesthesia (4:20).

**4:21** Both the primary neurosurgeon and ENT surgeon, if they’re right-handed, are on the right side of the patient and instruments are passed across the table. The primary monitor is facing neurosurgery and a secondary monitor faces ENT. The endoscope tower and the navigation towers are positioned at the head of the bed.

**4:43** Here is an intraoperative view of our exposure and the dural opening. As you can see, because of the extent of the tumor and its size (4:50), we had to drill slightly beyond the sphenoid limbus in order to expose some dura of the planum sphenoidale. You can already see a defect in the dura at the level of the tuberculum sella where the tumor is making strong attachments to the skull base to get its vascular supply. The dura is opened sharply using a round feather blade. The dural opening is expanded using microscissors all the way up to the sphenoid limbus (5:20); careful microsurgical circumferential dissection around the perimeter of the tumor is performed in order to maintain arachnoid planes (5:31) and preserve surrounding structures.

**5:35** Once the tumor is devascularized, the Sonopet is used to debulk the central core of the tumor, thus creating more space for dissection. Once more, space is created by further debulking; critical perforators (5:50) and surrounding neural structures like the optic nerve are carefully dissected off the perimeter of the tumor. These perpetrators are critical to preserve, as they may supply the optic chiasm and may lead to ischemic injury if they’re taken. Sharp microsurgical dissection is performed in areas where the tumor is adherent to the optic nerve. This prevents distortion (6:20) of the nerve as the tumor is being delivered from the resection bed. Tumor seen here is being delivered over the superior hypophyseal artery with careful care not to injure the artery as its distal branches may supply the pituitary stalk. Continued sharp, methodical microdissection will eventually lead to a tumor nidus that can be isolated and quarantined with cottonoids; once this is done (6:50), the Sonopet is used to debulk the remainder of the tumor till its distal attachments to the chiasm, which can be microdissected off using fine dissectors. These final attachments may be the strongest of all, and careful care must be done to remove this final remnant of tumor without injuring the optic chiasm. Residual tumor (7:20) seen here is carefully microdissected off using microscissors as the final piece of attachment of tumor to the chiasm. Careful inspection of the optic chiasm and pituitary stalk reveal intact vasculature as demonstrated here by ICG. Inspection of the surgical cavity reveals no residual tumor.

**7:53** Reconstruction involves a three-layer technique, which involves a DuraGen inlay to close off the dural defect. This is followed by a second layer, a fascia latta layer that is harvested from the right leg and followed by a third layer, the nasal septal flap, which has mobilized and positioned appropriately across the ventral skull base.

**8:18** The nasal septal flap (8:20) is then tacked up using Surgicel.

**8:27** Surgicel is used to maintain the perimeter of the flap and keep it well opposed to the skull base. Our team uses a lumbar drain that is opened postoperative day 1 and maintained for several days and then removed.

**8:46** Postoperative MRI demonstrated gross-total resection. An excellent decompression (8:50) of the optic apparatus. The patient returned back to her neurological baseline with improved vision out of her right eye but still no light perception in her left eye.
